# Shape-memory responses compared between random and aligned electrospun fibrous mats

**DOI:** 10.3389/fbioe.2023.1130315

**Published:** 2023-01-26

**Authors:** Xianliu Wang, Zhaowenbin Zhang, Chunping Qin, Xuran Guo, Yanzhong Zhang

**Affiliations:** ^1^ College of Biological Science and Medical Engineering, Donghua University, Shanghai, China; ^2^ Shanghai Engineering Research Centre of Nano-Biomaterials and Regenerative Medicine, Donghua University, Shanghai, China; ^3^ China Orthopaedic Regenerative Medicine Group (CORMed), Hangzhou, China

**Keywords:** shape-memory polymers, electrospinning, temperature memory effect, osteogenic differentiation, mechanoactive scaffold

## Abstract

Significant progress has been made in the design of smart fibers toward achieving improved efficacy in tissue regeneration. While electrospun fibers can be engineered with shape memory capability, both the fiber structure and applied shape-programming parameters are the determinants of final performance in applications. Herein, we report a comparison study on the shape memory responses compared between electrospun random and aligned fibers by varying the programming temperature *T*_*prog*_ and the deforming strain *ε*_*deform*_. A PLLA–PHBV (6:4 mass ratio) polymer blend was first electrospun into random and aligned fibrous mat forms; thereafter, the effects of applying specific *T*_*prog*_ (37°C and 46°C) and *ε*_*deform*_ (30%, 50%, and 100%) on the morphological change, shape recovery efficiency, and switching temperature *T*_*sw*_ of the two types of fibrous structures were examined under stress-free condition, while the maximum recovery stress *σ*_max_ was determined under constrained recovery condition. It was identified that the applied *T*_*prog*_ had less impact on fiber morphology, but increasing *ε*_*deform*_ gave rise to attenuation in fiber diameters and bettering in fiber orientation, especially for random fibers. The efficiency of shape recovery was found to correlate with both the applied *T*_*prog*_ and *ε*_*deform*_, with the aligned fibers exhibiting relatively higher recovery ability than the random counterpart. Moreover, *T*_*sw*_ was found to be close to *T*_*prog*_, thereby revealing a temperature memory effect in the PLLA–PHBV fibers, with the aligned fibers showing more proximity, while the *σ*_max_ generated was *ε*_*deform*_-dependent and 2.1–3.4 folds stronger for the aligned one in comparison with the random counterpart. Overall, the aligned fibers generally demonstrated better shape memory properties, which can be attributed to the macroscopic structural orderliness and increased molecular orientation and crystallinity imparted during the shape-programming process. Finally, the feasibility of using the shape memory effect to enable a mechanoactive fibrous substrate for regulating osteogenic differentiation of stem cells was demonstrated with the use of aligned fibers.

## Graphical Abstract

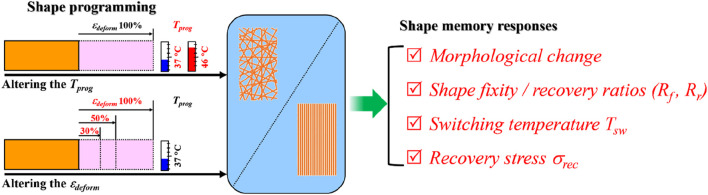

## 1 Introduction

Shape-memory polymers (SMPs) are highly morphologically responsive active materials (Huang et al., 2012), which are typically characterized by the shape recovery capability if having the polymers prior shape-programmed to any possible temporary shapes and later on stimulated to revert by applying certain environmental stimuli [e.g., heat (Lendlein and Kelch, 2002), light (Xie et al., 2018), and ultrasound (Bao et al., 2013)]. When the SMPs are produced in fibrous form and endowed with shape memory functionality by shape-programming, they give rise to a new type of functional fiber—shape-memory polymer fibers (SMPfs). Due to the intrinsic advantages in fibrous materials, such as high specific surface area, adjustable porosity, excellent mechanical properties, and structural designability, SMPfs have found a plethora of applications in textiles (Hu et al., 2012; Leist et al., 2017; Veloso-Fernandez et al., 2021), electronics (Dubal et al., 2018), aeronautics (Ahmed et al., 2021), and biomedical engineering, aimed for attaining enhanced functionalities (Lendlein et al., 2010; Zhao et al., 2019). Given that the shape memory performance of SMPs is closely correlated with their shape-memory creation procedure (SMCP) or shape-programming, an in-depth understanding on the influences of shape-programming parameters, in particular, the programming temperature *T*_*prog*_ and deforming (or programming) strain *ε*_*deform*_, on shape memory responses in SMPfs is essential.

Electrospinning has been widely recognized as one of the most efficient enabling nanotechnologies to produce ultrafine fibers at micro/nanoscale fineness. In the past decade, SMPfs from electrospinning have drawn a great deal of attention toward multifunctional applications, particularly in the fields of tissue engineering and regenerative medicine (Bao et al., 2014; Bao et al., 2016; Guo et al., 2022; L.-F. Tseng et al., 2013; Wang et al., 2020; Zhang et al., 2017). Just like routine SMPs, the performance of electrospun SMPfs is mainly defined by a few key shape-memory-responsive parameters, such as the shape fixity ratio (*R*_*f*_, the capability to fix the deformed shape) and shape recovery ratio (*R*_*r*_, the capability to recover the original shape), the switching temperature (*T*_*sw*_, the temperature above which the action of shape recovery takes place instantly), and the shape recovery stress (*σ*_*rec*_, the mechanical stress generated while the shape recovery action is activated under constrained recovery condition). Since electrospun fibers can be typically produced into random and aligned fibrous mat forms, a comparative study with respect to the shape-memory responses between the two types of fibrous structure is necessary, from which targeted applications with such SMPfs can be rationally designed.

**FIGURE 1:**
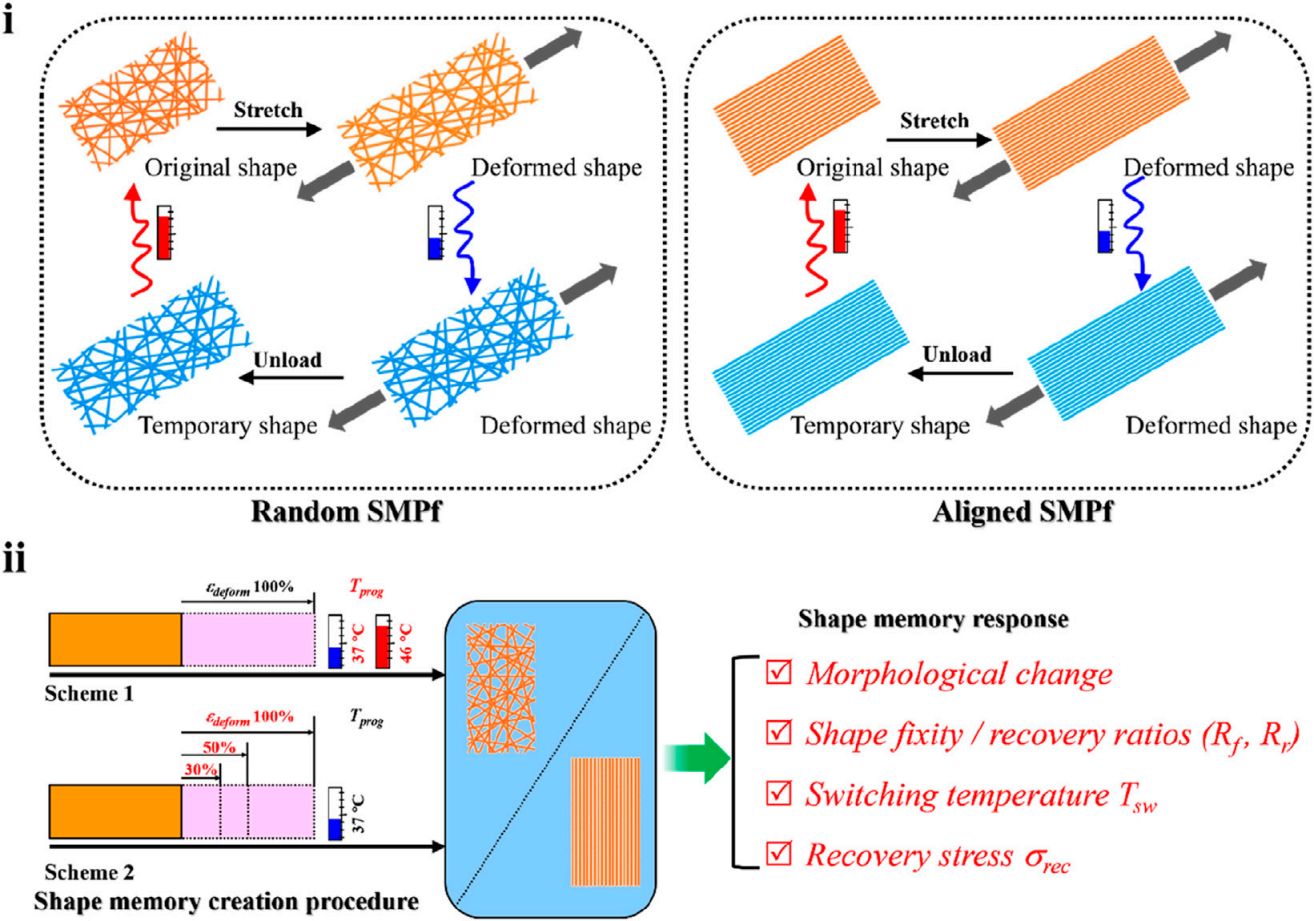
Study design overview. (i) Typical shape memory cycles including the SMCP and thermal-triggered shape recovery for the random (left) and aligned (right) fibrous mats. (ii) Comparative study on shape-memory responses including morphology change, shape fixity, recovery ratios (i.e., *R*_*f*_ and *R*_*r*_), switching temperature *T*_*sw*_, and recovery stress *σ*_*rec*_ regulated by two sets of programming schemes, namely, 1) altering the programming temperature *T*_*prog*_ (i.e., 37°C and 46°C) at the stretch deforming strain *ε*_*deform*_ of 100% and 2) altering *ε*_*deform*_ (i.e., 30%, 50%, and 100%) at *T*_*prog*_ of 37°C.

Previously, we have demonstrated that incorporation of a small amount of poly(3-hydroxybutyrate-*co*-3-hydroxyvalerate) (PHBV) into poly(_L_-lactic acid) (PLLA) fibers resulted in significant improvement on the shape memory performance of PLLA (Wang et al., 2021). Here, using the electrospun PLLA–PHBV hybrid system as a model fibrous material, we performed a comparative study on the shape-memory responses compared between the random and aligned fibrous mats while having them shape-programmed using two sets of programming schemes (i.e., varying *T*_*prog*_ or *ε*_*deform*_, Figure 1). Finally, the aligned fibrous mat of PLLA–PHBV was chosen to demonstrate the feasibility of using the integrated shape-memory effect (SME) to direct osteogenic differentiation of bone mesenchymal stem cells (BMSCs) *in vitro*.

## 2 Materials and methods

### 2.1 Electrospinning of random and aligned fibrous mats

The polymer blend of PLLA (M_v_ = 100,000 Da, DaiGang Biomaterial, Jinan, China) and PHBV (M_w_ = 520, 000 Da TianAn Biologic, Ningbo, China) at a mass ratio of 6:4 was electrospun into random fibrous mats (named random) and aligned fibrous mats (named aligned) by conventional electrospinning and stable jet electrospinning (SJES) methods (Yuan et al., 2012), respectively. In brief, 0.2 g of PHBV was added to 4.5 mL of trichloromethane (purity ≥99.0%, Changshu Yang-Park Chemicals, Changshu, China). The PHBV solution was subsequently heated to 65°C under stirring for 5 min, followed by adding 0.3 g of PLLA to obtain a homogeneous PLLA–PHBV solution. Last, a volume of 0.5 mL of N,N-dimethylformamide (purity ≥99.0%, Changshu Yang-Park Chemicals, Changshu, China) was introduced into the PLLA–PHBV solution to formulate the final polymer concentration of 10% w/v. Afterward, electrospinning was conducted as per the variables listed in Table 1. All the obtained fibrous mats were dried in vacuum at room temperature (23 ± 2°C).

**TABLE 1:** Parameters used for electrospinning aligned and random fibrous mats.

	Applied voltage (kV)	Solution feed rate (mL/h)	Collecting distance (cm)	Drum rotating speed (rpm)	Ambient temperature (°C)/humidity (%)
Aligned	7–9	0.5	3–6	1,000	20–30/40–50
Random	14–16	1	15–20	—	20–30/40–50

### 2.2 Characterization of the as-electrospun random and aligned fibrous mats

#### 2.2.1 Scanning electron microscopy

Surface morphology of the fibrous mats was observed by SEM (scanning electron microscopy) (FEI Quanta 250, Czech) at an accelerating voltage of 10 kV. Prior to imaging, the samples were subjected to sputter coating with a thin layer of gold for better conductivity. Upon acquiring the SEM images, fiber diameters were measured directly from the images using the ImageJ software, and 2D fast Fourier transform (FFT) was used to analyze the images and quantify the degree of fiber orientation through an oval profile plug-in (Ayres et al., 2008).

#### 2.2.2 Polarized Fourier transform infrared spectroscopy

The molecular orientation of the two types of fibrous mats was assessed by P-FTIR (polarized Fourier transform infrared spectroscopy) (Nicolet NEXUS 670 FTIR spectrometer, Thermo Fisher Scientific) (Wang et al., 2006), in which a PerkinElmer polarized wire grid over the range of 800–2,000 cm^−1^ at a scanning resolution of 2 cm^−1^ was used. Dichroic ratio (*D*_*r*_) for the specific absorption bands of the molecular chains within the PLLA–PHBV constituents can be calculated by Formula 1:

$$D_{r}=\frac{A_{//}}{A_{⊥}},$$ (1)

where A_//_ and A_⊥_ represent the peak absorption of infrared radiation polarized parallel and perpendicular to the fiber direction, respectively.

#### 2.2.3 X-ray diffraction

The crystalline structure of the two types of fibrous mats was examined by an X-ray diffractometer (Rigaku, Japan) using Cu–Kα radiation. Crystallinity was calculated from the ratio of the integrated area of all crystalline peaks to the total integrated area under the XRD (X-ray diffraction) peaks after background subtraction.

#### 2.2.4 Tensile test

The tensile properties of the two types of fibrous mats were assessed using a universal material testing machine (H5K-S, Hounsfield, United Kingdom) equipped with a 50-N load cell. A constant strain rate of 50%/min was used to stretch the samples at room temperature (n = 5). Ultimate tensile strength, Young’s modulus, and fracture strain were determined from the generated stress−strain curves.

#### 2.2.5 Dynamic mechanical analysis

Phase transition temperatures of the two types of fibrous mats were detected by DMA (dynamic mechanical analysis) (Q800, TA Instruments) in the multi-frequency strain mode. Briefly, rectangular fibrous mat samples (dimension: 10 × 5 × 0.06 mm) were stretched in the temperature sweep mode (applied oscillation frequency: 10 Hz) from −50 to 120°C with a constant heating rate of 1°C min^−1^. Phase transition temperatures can be scrutinized and accordingly determined at the peak maximum of the tan δ *vs*. temperature curves.

### 2.3 Examination of the SME-resultant morphological changes and mechanostructural properties

Rectangular fibrous samples (named Original dimension: 20 × 10 × 0.06 mm) were divided into different groups and deformed by stretching in warm water (37°C and 46°C) to the predesignated strain (*ε*_*deform*_ = 30%, 50%, and 100%), followed by shape-fixing without releasing the applied stress in a 4°C refrigerator (thus, deformed samples were named deformed). The so-programmed fibrous samples can be actuated to recover in warm water (37°C and 46°C); thus, recovered samples were named recovered. Fiber morphology, structural analysis, and tensile properties of the original, deformed, and recovered samples were examined as follows.

#### 2.3.1 Morphological observation

Morphological features including mainly fiber diameter and fiber orientation (defined as the degree to which fibers deviated from the tensile-loading direction) of the original, deformed, and recovered samples were similarly examined through SEM imaging as performed in 2.2.1 and quantitatively analyzed by the ImageJ software.

#### 2.3.2 Structural analysis

To examine the effect of shape-programming on the crystalline structure within the polymer fibers, the diffraction peaks and crystallinity of the original and deformed (*ε*_*deform*_ = 30%, 50%, and 100%) samples were similarly examined through XRD as performed in 2.2.3.

#### 2.3.3 Tensile test

The original and deformed (*ε*_*deform*_ = 30%, 50%, and 100%) samples were stretched at a strain rate of 50%/min by the aforementioned testing machine equipped with a 50-N load cell at room temperature. Young’s modulus can be computed accordingly from the generated stress−strain curves.

### 2.4 Determination of shape recovery ability by DMA

Shape recovery ability under the variation of pre-designated programming parameters (i.e., *T*_*prog*_ and *ε*_*deform*_ shown in Figure 1) was assessed by DMA. In general, each test cycle was consisted of three steps in sequence: stretching to deform into a temporary shape at the deforming temperature, having the deformed shape fixed at low temperature, and applying thermal stimulus to trigger the shape recovery. Specific procedures for performing the shape memory tests regulated by *T*_*prog*_ and *ε*_*deform*_ are depicted as follows.

Shape memory tests regulated by T_prog_: 1) At a high temperature *T*_*high*_ of 60°C, the fibrous mat sample was stretched using a strain ramp rate of 2%/min to 10% strain (*ε*_*begin*_); 2) the temperature was cooled down from *T*_*high*_ to *T*_*prog*_ (i.e., 37°C or 46°C) at 3°C/min; 3) at *T*_*prog*_, the sample was stretched to 100% strain (*ε*_*deform*_) at a ramp rate of 4%/min; 4) at the 100% strain, the temperature was decreased to a low temperature *T*_*low*_ of 0°C at 3°C/min for vitrification; 5) at 0°C, the applied stress was released to 0, from which the fixed strain (*ε*_*fix*_) can be obtained; and 6) the recovered strain (*ε*_*final*_) was determined by reheating the temperature to the *T*_*high*_ of 60°C for activating shape recovery. Based on the generated strain−temperature curves, a particular *T*_*sw*_ can be determined by finding the inflection point (corresponding to the maximum recovery speed) through differentiation, and *R*_*f*_ and *R*_*r*_ were calculated according to Formulas 2, 3, respectively:

$$R_{f}\left(\%\right)=\frac{\varepsilon_{fix}-\varepsilon_{begin}}{\varepsilon_{deform}-\varepsilon_{begin}}\times 100\%;$$ (2)

$$R_{r}\left(\%\right)=\frac{\varepsilon_{deform}-\varepsilon_{final}}{\varepsilon_{deform}-\varepsilon_{begin}}\times 100\%.$$ (3)

*Shape memory tests regulated by ε*_*deform*_: 1) At *T*_*high*_ of 60 °C, the fibrous mat sample was stretched at 2%/min to 10% strain (*ε*_*begin*_); 2) the temperature was cooled down from *T*_*high*_ to *T*_*prog*_ of 37°C at 3°C/min; 3) at 37°C, the sample was stretched using a strain ramp rate of 4%/min to *ε*_*deform*_ of 30%, 50%, or 100%; 4) at the same designated strain, the temperature was decreased to the *T*_*low*_ of 0°C at 3°C/min for vitrification; 5) at 0°C, the stress was released to 0, from which the fixed strain *ε*_*fix*_ was obtained; and 6) the sample was reheated to *T*_*prog*_ of 37°C and then maintained for *ca.* 3 h, during which a plot depicting the kinetics of deformed strain versus time can be generated. Normalization to 100% strain percentage was performed for the cases where *ε*_*deform*_=30%, 50%, and 100%.

### 2.5 Determination of the shape recovery stress

Similar to the procedure described in **2.3**, the fibrous mat samples deformed to the predesignated strain (*ε*_*deform*_ = 10–100% at a 10% interval) were prepared in 37°C warm water, followed by shape-fixing in a 4°C refrigerator. Thereafter, upon being triggered to recover in 37°C warm water, the maximal recovery stress *σ*_max_ of the deformed samples with their two ends fixed by clamps can be measured using a 5-N force gauge.

### 2.6 Proof-of-concept biological test

#### 2.6.1 Cell culture

Primary rat BMSCs were isolated from the bone marrow of 4-week-old male SD rats with the approval of the Animal Ethics Committee of Donghua University (No. 20140022). The obtained BMSCs were cultured in α-MEM supplemented with 10% (v/v) fetal bovine serum (FBS) and 1% (v/v) penicillin–streptomycin solution. The culture medium was refreshed every 2 days.

#### 2.6.2 Cell culture involving the *in situ* applied SME

BMSC-laden aligned fibrous scaffolds, prior sterilized by ethanol for 2 h and medium soaking overnight, were placed in a 6-well plate with 2 mL medium per well for 1 day of cultivation and then subjected to shape-programming in a Bose ElectroForce BioDynamic 5200 multi-chamber bioreactor. In brief, the cell-seeded fibrous scaffold was stretched in 37°C culture medium to 10% strain for 30 min (named Aligned 10%), followed by shape-fixing in 25°C culture medium for 1 h. Then, the temperature was increased to 37°C to trigger constrained shape recovery (Cui et al., 2011; Guo et al., 2022) in the scaffold for 4 h. Afterward, the cell–scaffold construct was moved to a 6-well plate for continuous culturing under stress-free condition. The previously described process was repeated at 3, 5, and 7 days. For comparison, the aligned fibrous scaffold seeded with BMSCs for cultivation but without undergoing the previously mentioned shape-programming process was used as a control and named Aligned 0%.

#### 2.6.3 Cell morphology

BMSCs were seeded onto the fibrous scaffolds at a density of 1 × 10^5^ cells/well in a 6-well plate. After 4 and 8 days of culture, the cell–scaffold constructs were washed three times with PBS and fixed in 2.5% glutaraldehyde at 4°C overnight. After being washed three times in distilled water, the constructs were dehydrated with gradient alcohol concentrations (25%, 50%, 75%, 85%, 95%, and 100% for 15 min each), followed by immersion in tertiary butanol for 10 min. Afterward, the constructs were freeze-dried and coated with gold for morphological observation by SEM.

#### 2.6.4 Cell proliferation

BMSCs were seeded onto the fibrous scaffolds at a density of 1 × 10^5^ cells/well in a 6-well plate. Cell proliferation was monitored by performing MTT (3-(4,5-dimethylthiazol-2-y1)-2,5-diphenyltetrazolium bromide) assay after 1, 4, 8, and 12 days of cultivation. Briefly, the medium was removed to have the constructs washed with PBS three times, and then 1 mL of medium and 100 μL of MTT (5 mg/mL) were added to each well. The plate was then incubated for 4 h, followed by removing the mixed solution of medium and MTT, adding 1 mL of dimethyl sulfoxide to each well, and pipetting up and down to dissolve crystals in dark. Finally, the solutions were transferred to a microplate reader (MK3, Thermo Fisher Scientific, USA) for absorbance measurements at 570 nm.

#### 2.6.5 Alkaline phosphatase activity

BMSCs were seeded onto the fibrous scaffolds at a density of 1 × 10^5^ cells/well in a 6-well plate. At days 8 and 21, the constructs were trypsin-treated to detach the cells for seeding into 24-well plates for ALP (alkaline phosphatase) staining. Briefly, after being fixed in 2.5% glutaraldehyde for 10 min on ice, the cells were incubated in a mixture of nitroblue tetrazolium and 5-bromo-4-chloro-3-indolylphosphate working solution (Alkaline Phosphatase Staining Kit, Beyotime, Shanghai, China) for 20 min. The reaction was stopped by removing the working solution and rinsing with PBS. Stained cells were visualized using an inverted optical microscope (ECLIPSE Ti-S, Nikon, Japan).

#### 2.6.6 Quantification of calcium deposits

BMSCs were seeded onto the fibrous scaffolds at a density of 1 × 10^5^ cells/well in a 6-well plate. The Calcium (CPC) LiquiColor Test (StanBio, USA) was used to quantify the calcium deposition of cells cultured for 8, 14, and 21 days according to the manufacturer’s instructions. In brief, the constructs were washed with PBS (free of calcium and magnesium ions) and treated with 0.5 N hydrochloric acid, and then cells were scraped and collected into an appropriate centrifuge tube. After shaking for 3 h with an orbital shaker, the cells were centrifuged, and the supernatant was transferred into a new centrifuge tube followed by adding ortho-cresolphthalein complexone (OCPC). The optical absorbance was then measured using a microplate reader (BioTek, USA) at a wavelength of 550 nm.

#### 2.6.7 Gene expression analysis

BMSCs at a density of 1 × 10^5^ cells/well in a 6-well plate were seeded onto the fibrous scaffolds for 1, 8, and 21 days of culture. After total RNA extraction using TRIzol reagent (Invitrogen, USA), amplifications were performed with different primers (Table 2). The quality and quantity of the obtained RNA were subjected to spectrophotometric analysis using a bio-photometer (Thermo Scientific, NanoDrop 2000). The RNA was then reverse-transcribed onto complementary DNA (cDNA) using a reverse transcription kit (Takara, Japan). Quantitative real-time polymerase chain reaction (qPCR) was performed with the SYBR Green PCR reagent kit (Roche, Germany) on an ABI Prism 7500 (Applied Biosystems, USA). The comparative expression level (fold change) was obtained by transforming the logarithmic values into absolute values using the 2^−△△Ct^ method (Livak and Schmittgen, 2001).

**TABLE 2:** Primer sequences used for qRT-PCR gene expression analysis.

Gene	Forward primer sequence (5′-3′)	Reverse primer sequence (5′-3′)
*Runx2*	TCCGCCACCACTCACTA	GGACGCTGACGAAGTACC
*Alp*	CCG​CAG​GAT​GTG​AAC​TAC​T	GGT​ACT​GAC​GGA​AGA​AGG​G
*Ocn*	ACC​GAG​ACA​CCA​TGA​GAG​C	GCTGCACCTTTGCTGGA
*GAPDH*	TGG​AAT​TGT​GAG​GGA​GAT​G	GCCCAGCAAGGATACTGA

### 2.7 Statistical analysis

Quantitative data are generally presented as mean ± standard deviation and checked by normality tests. Statistical analysis was performed using the Origin software (OriginLab, Northampton, MA, USA). One-way ANOVA with *post hoc* Tukey’s HSD test was used to make pairwise comparisons between groups. *p < 0.05, **p < 0.01, or ***p < 0.001 is considered to be statistically significant.

## 3 Results and discussion

### 3.1 Characterization of the as-electrospun fibrous mats

**FIGURE 2:**
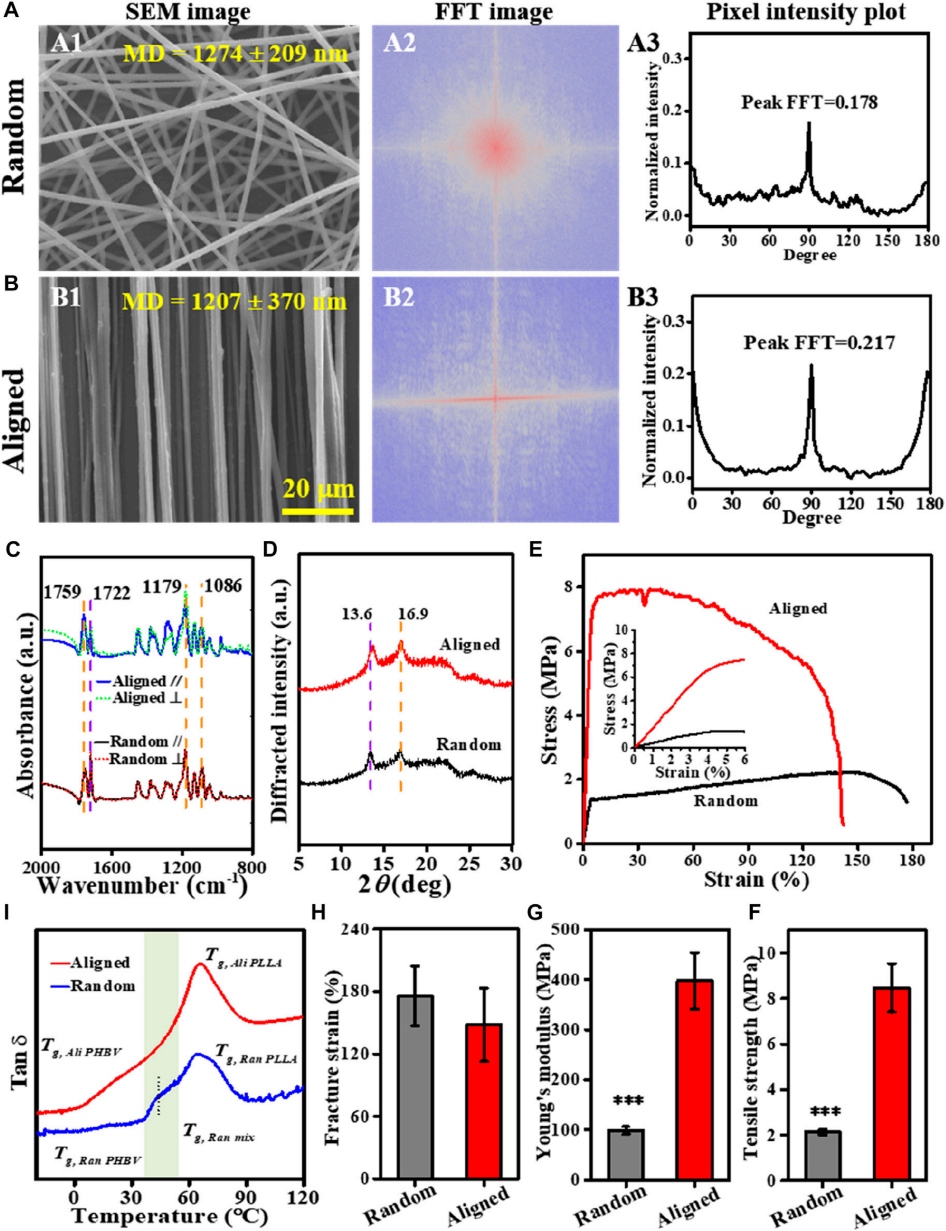
Characterization of the PLLA–PHBV based on random and aligned fibrous mats. **(A, B)** Alignment analysis of the electrospun fibers: SEM images (A1 and B1), FFT output images (A2 and B2), and pixel intensity plots (A3 and B3). **(C)** Polarized FTIR spectra [The black and blue lines are obtained with the beam polarized parallel to the fiber axis (A_//_), whereas the dotted red and green lines are obtained with the beam polarized perpendicular to the fiber axis (A_⊥_)]. **(D)** XRD patterns. **(E–H)** Tensile tests showing typical stress–strain curves, tensile strength, Young’s modulus, and fracture strain, respectively. **(I)** Tan δ−temperature curves *via* DMA.

Both random and aligned fibers of the PLLA–PHBV blend with comparable diameters (1,274 ± 209 nm *vs*. 1,207 ± 370 nm) could be readily produced using the conventional electrospinning and SJES methods, respectively (Figures 2A1, B1). The FFT output images (graphical depiction of FFT frequency, Figures 2A2, B2) and the derived pixel intensity plots (Figures 2A3, B3) indicate that, compared to the random group, a distinctly higher degree of fiber anisotropy is noted in the aligned group by showing concentrated pixels mainly in a cross-like style and a sharp peak (Ayres et al., 2006; Ayres et al., 2007). At the molecular level, the P-FTIR results (Figure 2C) show strong molecular orientation of the polymer chains within the aligned fibers, as there were pronounced differences in absorbance intensity in the directions parallel and perpendicular to the fiber alignment. This is true either for the characteristic C-O-C symmetric stretch at 1,086 cm^−1^, OC-O asymmetric stretch at 1,179 cm^−1^, and C=O vibration at 1,759 cm^−1^ in PLLA (Zhang et al., 2010) or the C=O stretch at 1,722 cm^−1^ ascribed to PHBV (Furukawa et al., 2005). However, the polarized measurements in the ⊥ and // directions were almost identical in the case of random fibers. The quantitative dichroic ratios (*D*_*r*_) with respect to the aligned group, showing values of higher deviation from the data of 1.00 (Supplementary Table S1), support the observed enhancement of molecular orientation in the aligned fibers (Chan et al., 2009; Wang et al., 2006). Such an enhancement in molecular orientation can be attributed to the SJES technique itself (Yuan et al., 2012; Zhang & Zhang, 2016) and the strong stretching exerted by the high-speed rotating drum. A higher extent of molecular orientation benefits the formation of more crystalline structures (Figure 2D). This was also evidenced by the appearance of reflection peaks at 2*θ* = 16.9 and 2*θ* = 13.6, corresponding to the (110) and (020) crystal planes of PLLA (Sun, Yu, Zhuang, Chen, & Jing, 2011; Zembouai et al., 2013) and PHBV (Zembouai et al., 2013; Montanheiro et al., 2019), respectively. Obviously, the characteristic peak intensities in the XRD pattern of the aligned group are higher than those in the random group. As anticipated, due to the macroscopic and molecular orientation, aligned fibers exhibited superior mechanical properties (Figures 2E–H) than the random counterpart. Despite a slightly lower fracture strain, the aligned fibers are about 4-fold stronger in tensile strength (i.e., 8.5 ± 1.1 *vs*. 2.1 ± 0.1 MPa, *p* < 0.001) and Young’s modulus (i.e., 397.4 ± 56.3 *vs*. 98.8 ± 7.6 MPa, *p* < 0.001) than the random fibers.

Thermal-responsive SMPs usually rely on the thermal transition (e.g., glass transition temperature *T*_*g*_) to determine the temperature window for shape-programming. Figure 2I shows the tan δ−temperature curves of the random and aligned fibers non-isothermally scanned *via* DMA. Clearly, either the random or the aligned fibers exhibited a pretty broad thermal transition region with the maximum peak at *ca*. 65°C, which can be ascribed to *T*_*g*_ of the dominant constituent PLLA (*T*_*g, PLLA*_) within the PLLA–PHBV blend (Nanda et al., 2011; Jaszkiewicz et al., 2013; Li et al., 2015). A further subtle scrutinization below *T*_*g, PLLA*_ revealed the presence of an indistinct peak around 18°C attributed to *T*_*g*_ of PHBV (*T*_*g, PHBV*_) (Nanda et al., 2011; Jaszkiewicz et al., 2013) and a relaxation process with the peak maximum at 43.7°C in the range from 36°C to 54°C, indicative of *T*_*g*_ of the mixed phase (*T*_*g, mix*_) (Nanda et al., 2011), especially in the case of random. Although the much higher *T*_*g, PLLA*_ does not allow adaptation of *T*_*sw*_ for shape recovery up to the physiologically relevant temperature, choosing *T*_*prog*_ in the vicinity of *T*_*g, mix*_ of PLLA–PHBV would afford an alternative strategy to easily adjust *T*_*sw*_ due to the well-documented temperature-memory effect (TME) (Miaudet et al., 2007; Kratz et al., 2011; Kratz et al., 2012). In this sense, 37°C and 46°C (slightly above body temperature) in the range of Δ*T*_*g, mix*_ for the mixed phase (i.e., 36–54°C) can be chosen as *T*_*prog*_ for our subsequent shape-memory response studies.

### 3.2 Effects of shape-programming parameters on fiber morphology

**FIGURE 3:**
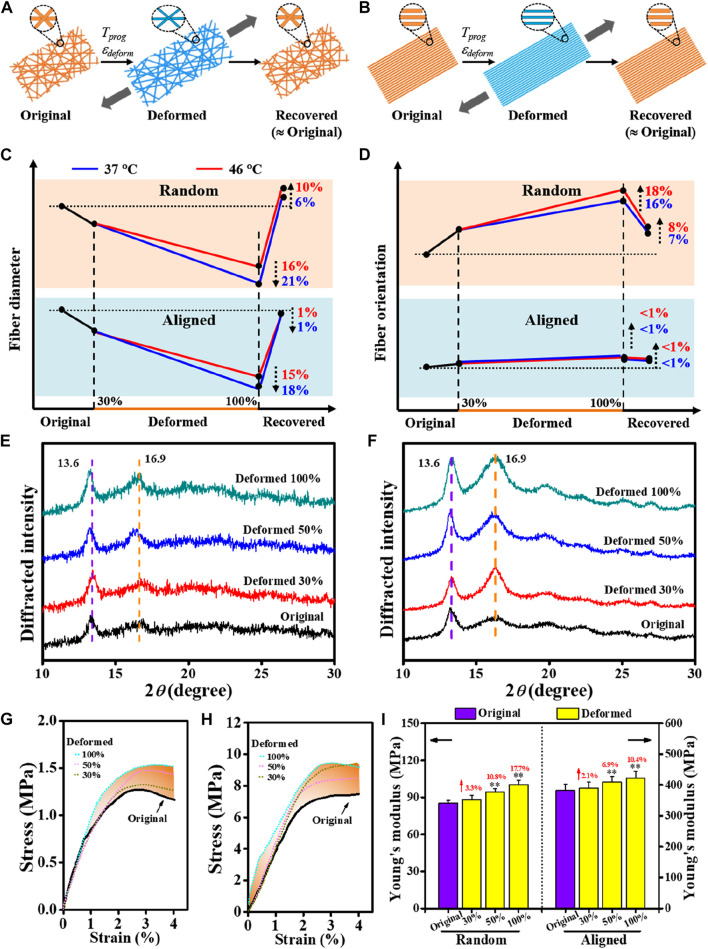
Comparative study on morphology changes regulated by shape-programming parameters between random and aligned fibers. **(A, B)** Schematics of morphology changes in a complete shape memory cycle. **(C, D)** Dynamic changes in fiber diameter and fiber orientation regulated by shape-programming. **(E, F)** XRD patterns due to applying *ε*_*deform*_ varied in 30%, 50%, and 100% at *T*_*prog*_ of 37°C **(E)** for random and **(F)** for aligned). **(G, H)** Representative tensile stress−strain curves of original and deformed (G for random and H for aligned). **(I)** Young’s moduli derived from **(G)** and **(H)**.

For shape-memory polymer fibers, stretching deformation to generate a temporary shape usually results in a reduction in fiber fineness together with improved fiber orientation along the loading direction; and upon being triggered for shape recovery, the morphological features of the original fibrous structure can be generally recovered (Figures 3A, B). This was verified in our current study by varying *T*_*prog*_ and *ε*_*deform*_. As shown in Figures 3C, D summarized from the SEM quantification data (Supplementary Figures S1A–F), under the deforming conditions of stretching in warm water (37°C and 46°C), for instance, 100% of *ε*_*deform*_, it gave rise to fiber diameter attenuation by 16–21% in random, slightly larger than that of aligned (15–18%). Also, a higher magnitude of improvement in fiber orientation was observed in the random group (16–18%) than in the aligned group (<1%). In terms of shape recovery, while the fiber thickness and orientation features of the aligned fibers could be largely reverted, small differences in morphological restoration can be noted in the random fibers.

Mechanistically, apart from the macroscopic differences in their fibrous architectures (Figures 2A, B), the initial status of molecular orientation (Figure 2C) and the stretching deformation resultant enhancement in crystallization may also be responsible for the different responses in the previously noted morphological changes. Taking the stretching deformation for shape-programming at 37°C for illustration (Figures 3E, F), due to the strain-induced crystallization effect (Tabatabaei et al., 2009; Tcharkhtchi et al., 2014; Nissenbaum et al., 2020), the increments of polymer crystallites formed in overall crystallinity at 100% of *ε*_*deform*_ could be increased by 22.9% and 110.9% (both mainly contributed by the PLLA crystalline phase, Table 3) for the random and aligned fibers, respectively. As a result, it also gave rise to the progressively strengthened elastic modulus and stiffness (Figures 3G–I; Supplementary Figure S2) if increasing *ε*_*deform*_ of both the random and aligned fibers during the stage of shape-programming. Collectively, these results demonstrated that the morphological changes were not dramatically influenced by *T*_*prog*_ but responded more to changes in *ε*_*deform*_. The stretching deformation and recovery process could, therefore, be applied to control the diameter and orientation of the shape-memory-capable electrospun fibrous structures, which may provide a dynamic topography for modulating the cellular behavior (Ahn et al., 2014; Madl et al., 2021; Song et al., 2013).

**TABLE 3:** Crystallinity of Deformed and Original measured by XRD for random and aligned.

Sample		*X*_*c*__*PLLA*_ (%)	*X*_*c*__*PHBV*_ (%)	*X*_*c*__*Overall*_ (%)
Random	Original	1.3	3.4	4.8
	30% deform	1.7	3.4	5.1
	50% deform	2.0	3.3	5.3
	100% deform	2.0	3.8	5.9
Aligned	Original	3.7	6.4	10.1
	30% deform	6.2	6.1	12.3
	50% deform	7.8	6.2	14.0
	100% deform	14.5	6.8	21.3

### 3.3 Effects of shape-programming parameters on *T*_*sw*_, *R*_*f*_, *and R*_*r*_

**FIGURE 4:**
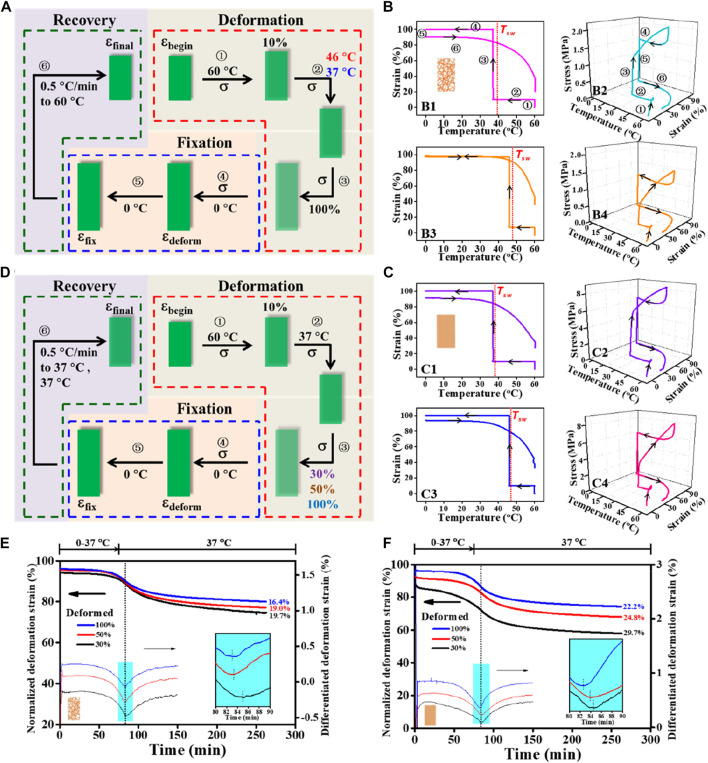
Comparative study on shape fixity, recovery ratios (i.e., *R*_*f*_ and *R*_*r*_), and switching temperature *T*_*sw*_ regulated by *T*_*prog*_ and *ε*_*deform*_ between random and aligned fibers. **(A)** Schematic representation of a cyclic shape memory test protocol consisted of stretching deformation (*T*_*prog*_ varied at 37°C and 46°C; *ε*_*deform*_ = 100%), fixation, and recovery stages under stress-free condition. **(B, C)** Two-dimensional illustrations of strain−temperature and three-dimensional diagrams of stress−strain−temperature for random **(B)** and aligned **(C)** tested using the protocol depicted in **(A)**; *T*_*prog*_ used for B1, B2, C1, and C2 is 37°C and for B3, B4, C3, and C4 is 46°C. **(D)** Schematic representation of a cyclic shape memory test protocol consisted of stretching deformation (*ε*_*deform*_ varied at 30%, 50%, and 100%; *T*_*prog*_ = 37°C), fixation, and recovery stages under the stress-free condition. **(E, F)** Kinetics of the recovery process for random **(E)** and aligned **(F)** tested using the protocol depicted in **(D)**.

TME is the capability of a shape-memory polymer to memorize its thermomechanical history, in particular the *T*_*prog*_ at which it was deformed before, by reversing the deformation at a characteristic *T*_*sw*_ roughly identical to the previously applied *T*_*prog*_. While the TME concept has been previously demonstrated in different material systems (Cui et al., 2011; Kratz et al., 2011; Miaudet et al., 2007; Xie et al., 2011), how well *T*_*sw*_ coordinates with *T*_*prog*_ in SMPfs remains to be understood. It has been known that temperature memory polymers usually exhibit a broad thermal transition temperature region *ΔT*_*trans*_ within which the *T*_*prog*_ applied for deformation can be memorized (Cui et al., 2010; Cui et al., 2011) during a specific SMCP. Based on the identified Δ*T*_*g, mix*_ for the mixed phase (Figure 2I), the effects of applying *T*_*prog*_ with physiological relevance (37°C and 46°C) at 100% of *ε*_*deform*_ on *T*_*sw*_, *R*_*f*_, and *R*_*r*_ were first examined (Figure 4A). A quantitative summary on the comparison of shape-memory properties between random and aligned (Table 4) indicates an impressive fixation of the deformation with *R*_*f*_ > 95.0, while *R*_*r*_ > 70% could be obtained. It is to be noted that the characteristic *T*_*sw*_, defined by the temperature with the maximum recovery speed under stress-free conditions, was always slightly higher than *T*_*prog*_ (Figures 4B, C). As a proximity indicator, the much lower *T*_*sw*_/*T*_*prog*_ ratios of 1.02–1.06 compared to other forms of SMPs (e.g., films) with *T*_*sw*_/*T*_*prog*_ ≈ 1.3 at 37°C reported in the literature (Cui et al., 2010) suggest superior thermal sensitivity of the fibrous structures due to their intrinsic attribute of high specific surface area (Lu et al., 2017). When making a comparison between random and aligned, the characteristic *T*_*sw*_ is closer to the applied *T*_*prog*_ in aligned. This can be ascribed to the better thermal conductivity in aligned fibers with structural features that include not only the high degree of macroscopic fiber alignment (Figures 2A, B) but also the increased molecular orientation and crystallinity (Figures 3E, F) (Shen et al., 2010; Lu et al., 2017; Bai et al., 2018). These results confirmed that the characteristic *T*_*sw*_ of TME can be more accurately correlated with *T*_*prog*_ for shape-memory polymer fibers, which could therefore permit a facile adjustment of *T*_*sw*_ according to specific biological application requirements (Kratz et al., 2012).

**TABLE 4:** Shape-memory properties of random and aligned determined in cyclic shape memory tests by DMA.

	*T*_*prog*_ (°C)	*ε*_*begin*_ (%)	*ε*_*deform*_ (%)	*ε*_*fix*_ (%)	*ε*_*final*_ (%)	*R*_*f*_ (%)	*R*_*r*_ (%)	*T*_*sw*_ (°C)	*T*_*sw*_ */T*_*prog*_
Random	37	10	100	96.8	32.1	96.4	75.4	39.4	1.06
	46	10	100	98.1	36.0	97.9	71.1	47.7	1.04
Aligned	37	10	100	96.0	32.1	95.6	75.4	38.2	1.03
	46	10	100	97.2	36.4	96.9	70.7	47.0	1.02

To examine whether varying *ε*_*deform*_ (30%, 50%, and 100%) would regulate the recovery ability of the two types of fibrous structures at body temperature, a further comparison between random and aligned fibers was then performed (Figure 4D). As shown in Figures 4E, F, depicting the recovery kinetics for random and aligned which were prior-deformed at different *ε*_*deform*_, and upon being triggered at *T*_*prog*_ of 37°C, the shape recovery events can be activated for both. Notably, occurrences of the maximum recovery rates determined by differentiation to the normalized *ε*_*deform*_ curves in aligned fibers were shorter than those in random ones (aligned 8.5–10.7 min *vs*. random 9–11 min), and the recovered strain of aligned fibers during the testing time frame was always higher than that of random counterparts at the same *ε*_*deform*_. Again, the observed better shape recovery performance in aligned fibers can be attributed to its high thermal conductivity and the underlying conducive molecular structures in connection. Consistent with previous investigations (Wong and Venkatraman, 2010; Wu et al., 2018; Zhao et al., 2017), in both cases, fibrous structures with increased *ε*_*deform*_ during the SMCP gave rise to decreased shape recovery efficiency.

### 3.4 Effect of deforming strain on recovery stress

**FIGURE 5:**
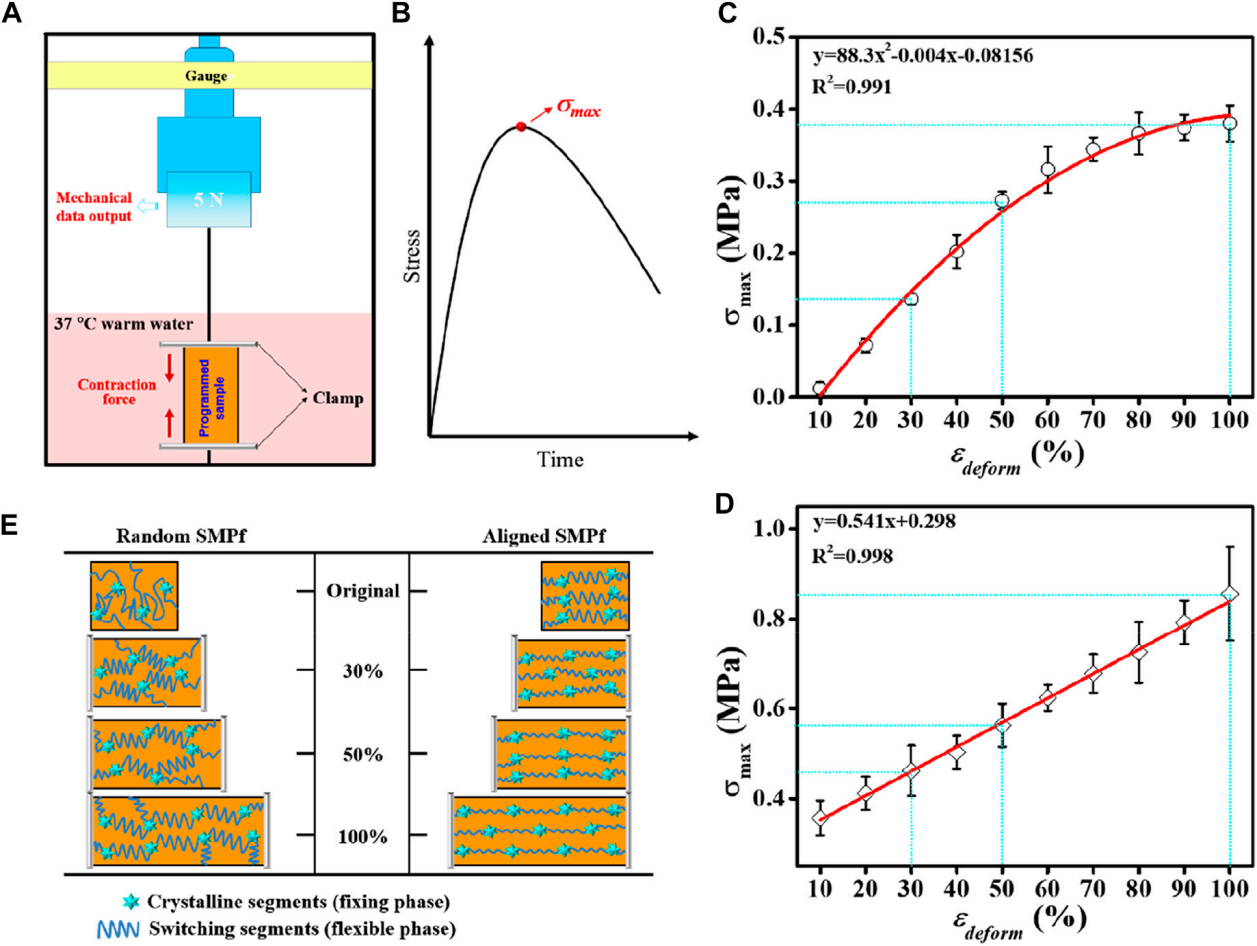
Comparative study on the shape recovery stress *σ*_*rec*_ regulated by the deforming strain *ε*_*deform*_ between random fibers and aligned fibers. **(A)** Schematic representation of the test device for *σ*_*rec*_ measurements. **(B)** Schematic representation of the stress−time curve from the constrained recovery test in **(A)**, with the peak stress indicating *σ*_max_. **(C, D)** Relationships of the maximum recovery stress (*σ*_max_) versus ε_*deform*_ determined from **(A)** for random **(C)** and aligned **(D)**, respectively. **(E)** Proposed mechanism of PLLA–PHBV shape-memory fibers using a stretch deforming process with the varied *ε*_*deform*_ (30%, 50%, and 100%).

The shape recovery process upon being triggered is usually accompanied by the release of the strain energy stored during deformation, which accordingly enables the generation of recovery stress (*σ*_*rec*_) and other shape-memory responses. To compare the impact of varying *ε*_*deform*_ on the generated *σ*_*rec*_ between random and aligned, a pair of custom-made grips was used to fasten the fibrous mat sample for *σ*_*rec*_ measurement under constrained recovery condition (Figure 5A). This allowed producing a stress versus time curve showing a maximum stress point (*σ*_max_) (Figure 5B). The presence of such a peak recovery stress under constrained recovery condition has also been observed previously for other SMPs (Cui et al., 2010; Kratz et al., 2011; Kratz et al., 2012). By plotting the *σ*_max_ versus ε_*deform*_ graphs (Figures 5C, D), it was revealed that *σ*_max_ could be systematically adjusted by variation of *ε*_*deform*_, and the *σ*_max_ versus ε_*deform*_ correlations for random and aligned could be mathematically well-fitted in polynomial and linear regressions, respectively. As the stress generated by shape recovery is a growing function of the strain energy stored during deformation at a high temperature (Miaudet et al., 2007), it is reasonable to observe continuous increases in *σ*_max_ with increasing *ε*_*deform*_. In comparison, *σ*_max_ generated by aligned is significantly higher than that generated by the random counterpart. Specifically, when the two types of fibrous mats with 30%, 50%, and 100% *ε*_*deform*_ were recovered in 37°C warm water, the *σ*_max_ generated from aligned was correspondingly 3.4-fold, 2.1-fold, and 2.3-fold stronger than that generated by random.

For the thermal-responsive SMP systems on the molecular level, it is the netpoints (e.g., crystallites, rigid segments, or chemical crosslinks that determine the permanent shape) and the switching segments (e.g., amorphous fractions in a polymer acting as a reversible phase) within the SMPs that constitute the two structural elements responsible for the SME (Salaris et al., 2022). Also, applying a stretch deforming strain *ε*_*deform*_ at a high temperature (i.e., *T*_*prog*_) and then cooling down cause the polymer chains to become oriented and trapped in the temporarily fixed shape, thus storing strain energy. When being reheated in the vicinity of *T*_*trans*_ (e.g., *T*_*g*_) associated with the switching domains, the polymer chains become mobile enough to move back to their random coil-like conformation, thus leading to recovery of the original shape. Since the *σ*_max_ generated during the shape recovery process is associated with the netpoint/switching segment density or hard segment weight content (Cui et al., 2010; Kratz et al., 2012) and considering the fact that increasing *ε*_*deform*_ by stretching gave rise to the polymer chains highly oriented (Yano et al., 2012; Youm et al., 2016) accompanying with the formation of more crystallites (Figure 3F), the observed higher recovery stress in aligned than in random can be reasonably explicated as illustrated in Figure 5E.

### 3.5 SME regulated osteogenic differentiation

**FIGURE 6:**
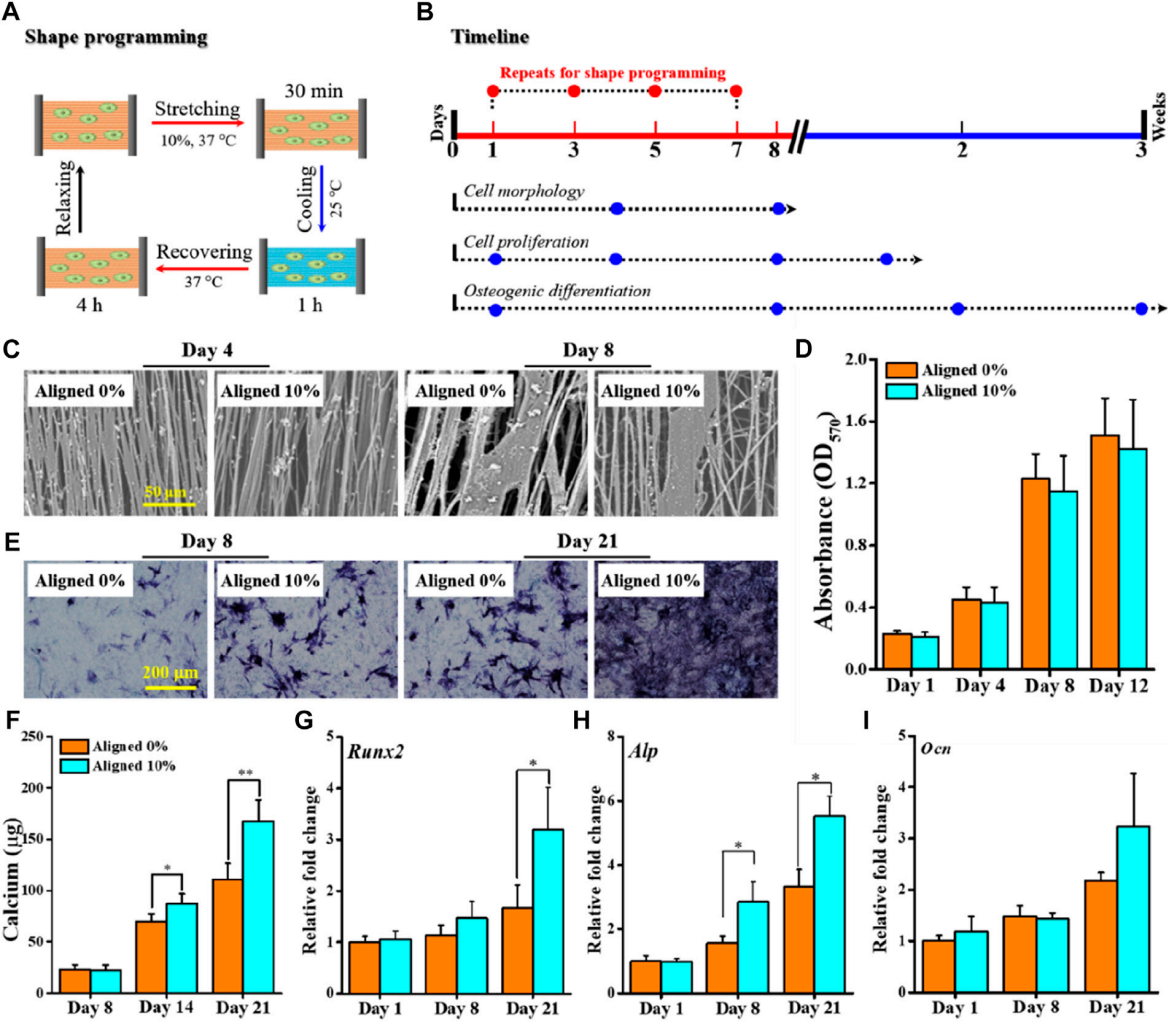
Proof-of-concept test of applying aligned fibrous substrate-based SME for regulating osteogenic differentiation. **(A)** Schematic representation of a complete cycle of shape-programming and recovery processes applied for *in vitro* cell culture with BMSCs. **(B)** Timeline depicting the process of cell culture and the designated biological assays to be performed at the specified time points. **(C)** SEM images of BMSCs cultured for 4–8 days; scale bar is 50 μm. **(D)** Histogram of BMSC proliferation by the MTT assay. **(E, F)** Osteogenic differentiation evaluated by ALP staining **(E)** and quantification of calcium deposits **(F)**. **(G–I)** Gene expression levels of representative osteogenic markers *Runx2* **(G)**, *Alp* **(H)**, and *Ocn* **(I)** at 1, 8, and 21 days.

The previously presented results suggest that the TME allows us to judiciously choose *T*_*prog*_ from *ΔT*_*trans*_ for shape-programming and triggering shape recovery at *T*_*sw*_ with physiological relevance (e.g., 37°C) and that the shape recovery stress generated under constrained recovery condition may be utilized to endow the fibrous scaffolds with mechanoactivity for *in situ* modulating cellular behavior (e.g., osteogenic differentiation of stem cells). To prove this concept, an aligned fibrous mat was chosen for stretch deforming the aligned fibers to 10% of *ε*_*deform*_ (without affecting cell survival (Altman et al., 2001)) at *T*_*prog*_ of 37°C, from which a mechanically dynamic culture system based on the SME-enabled mechanoactive fibrous scaffold can be constructed to direct the fate of BMSCs (Figure 6A). Cytocompatibility assessments based on the planned experimental scheme (Figure 6B) indicated that both the plain aligned 0% and the mechanoactive aligned 10% could support BMSCs to attach and grow along the fiber direction (Figure 6C), and the cell proliferation capacities of both were comparable during the 12 days of observation (Figure 6D). These results suggest that the integrated dynamic shape-programming and recovery processes during cell culture had no detrimental effect on cell growth, which is in good accordance with previous observations (Guo et al., 2022; Tseng et al., 2016; Xing et al., 2016).

Both the ALP staining and calcium quantification assay were performed to evaluate the efficacy of using the mechanoactive scaffold for promoting osteogenic commitment of BMSCs in the absence of any osteogenic induction factors. Obviously, the cells on the mechanoactive aligned 10% stained positively for endogenous ALP activity, whereas the expression of ALP in cells cultured on the aligned 0% was comparably quite low during the 21 days of culture (Figure 6E). The quantified content of calcium deposits, as another key indicator of osteogenic differentiation of BMSCs, similarly revealed statistically significant higher levels of calcium production in the aligned 10% group than in the aligned 0% group after 14 and 21 days of culture (Figure 6F). At the gene level, expressions of representative osteogenic markers including *Runx2*, *Ocn*, and *Alp* were also detected (Figures 6G–I). The mRNA expression patterns generally show that while the early time of expression (e.g., at days 1 and 8) is comparable, higher levels of markers at day 21 can be detected. These results consistently corroborated that applying *in situ* mechanical cues provided by the SME-enabled mechanoactive scaffold *per se* indeed resulted in achieving enhanced efficiency in directing osteogenic differentiation of stem cells.

In bone tissue engineering, applying appropriate means to efficiently drive osteodifferentiation of mesenchymal stem cells (MSCs) is crucial to osteogenesis. While utilization of various potent osteogenic factors (e.g., bone morphogenetic protein, dexamethasone, and transforming growth factor) has been demonstrated to be effective, given the mechanosensitive nature of bone, an attractive strategy for regulating osteogenic differentiation of MSCs has been the use of mechanical stimuli. Amongst, substrate stiffness has been identified to play a key role in directing stem cell osteodifferentiation (Gazquez et al., 2016; Lee et al., 2018; Song et al., 2021). In our current study, since shape recovery under constrained recovery condition can generate a stress-stiffening effect in the fibrous substrate (Guo et al., 2022), directing stem cell differentiation into an osteogenic lineage based on the SME-enabled mechanoactive scaffold itself, rather than using external bioreactors for exerting mechanical stimulus, was proved to be successful. The feasibility demonstrated here provided a fresh paradigm for developing mechanically active scaffolds for regulation of osteogenic differentiation in MSCs without using any biological supplements and/or bioreactors to provide osteogenic induction cues.

## 4 Conclusion

In summary, a systematic comparison of shape-memory responses between random and aligned electrospun fibrous mats was successfully performed by varying *T*_*prog*_ (37°C and 46°C) and *ε*_*deform*_ (30%, 50%, and 100%) applied during the SMCP. Compared to the applied *T*_*prog*_, increasing the *ε*_*deform*_ was found to have a more pronounced influence on the fiber diameters and fiber orientation, especially for the random one. The efficiency of shape recovery was *T*_*prog*_- and *ε*_*deform*_-dependent, with the aligned fibers exhibiting better recovery capability than random fibers. The *σ*_max_ generated in aligned was 2.1–3.4 folds stronger than that generated in random. The fibrous PLLA–PHBV was observed to possess a temperature memory effect as the determined characteristic *T*_*sw*_ was found to be close to the applied *T*_*prog*_. This distinctive temperature memory feature provides a possibility to select a physiologically relevant temperature for applications in the tissue engineering setting. The demonstrated feasibility of the shape memory fibrous substrate for osteogenic differentiation exploited the shape recovery stress controlled at a physiologically relevant temperature as an *in situ* exerted mechanical stimulus for modulation of stem cell fate, without using any biological or other mechanical induction cues. This proof-of-concept study may, therefore, provide a new paradigm in engineering mechanoactive scaffolds for promoting cellular functions to achieve desired outcomes in tissue regeneration.

## Data Availability

The original contributions presented in the study are included in the article/Supplementary Material; further inquiries can be directed to the corresponding author.
