# Supplementary material for: Shape-memory responses compared between random and aligned electrospun fibrous mats
Source: Front Bioeng Biotechnol. 2023 Jan 26;11:1130315. doi: 10.3389/fbioe.2023.1130315 (PMC9909598; doi:10.3389/fbioe.2023.1130315)
Supplement: Supplementary file 1 [file DataSheet1.docx]

Supplementary material

**Shape-Memory Responses Compared between Random and Aligned Electrospun Fibrous Mats**

Xianliu Wang^†^, Zhaowenbin Zhang^†^, Chunping Qin^†^, Xuran Guo^†^, and Yanzhong Zhang^†, ‡, §^*

^†^College of Biological Science and Medical Engineering, Donghua University,

Shanghai 201620, China

^‡^Shanghai Engineering Research Centre of Nano-Biomaterials and Regenerative Medicine,

Donghua University, Shanghai 201620, China.

^§^China Orthopaedic Regenerative Medicine Group (CORMed), Hangzhou 310058, China

*Corresponding author.

Yanzhong Zhang, Ph.D., Professor of Biomaterials, College of Biological Science and Medical Engineering, Donghua University, 2999 North Renmin Road, Shanghai 201620, China. Tel/Fax: +86 21 6779 2374, Email: yzzhang@dhu.edu.cn


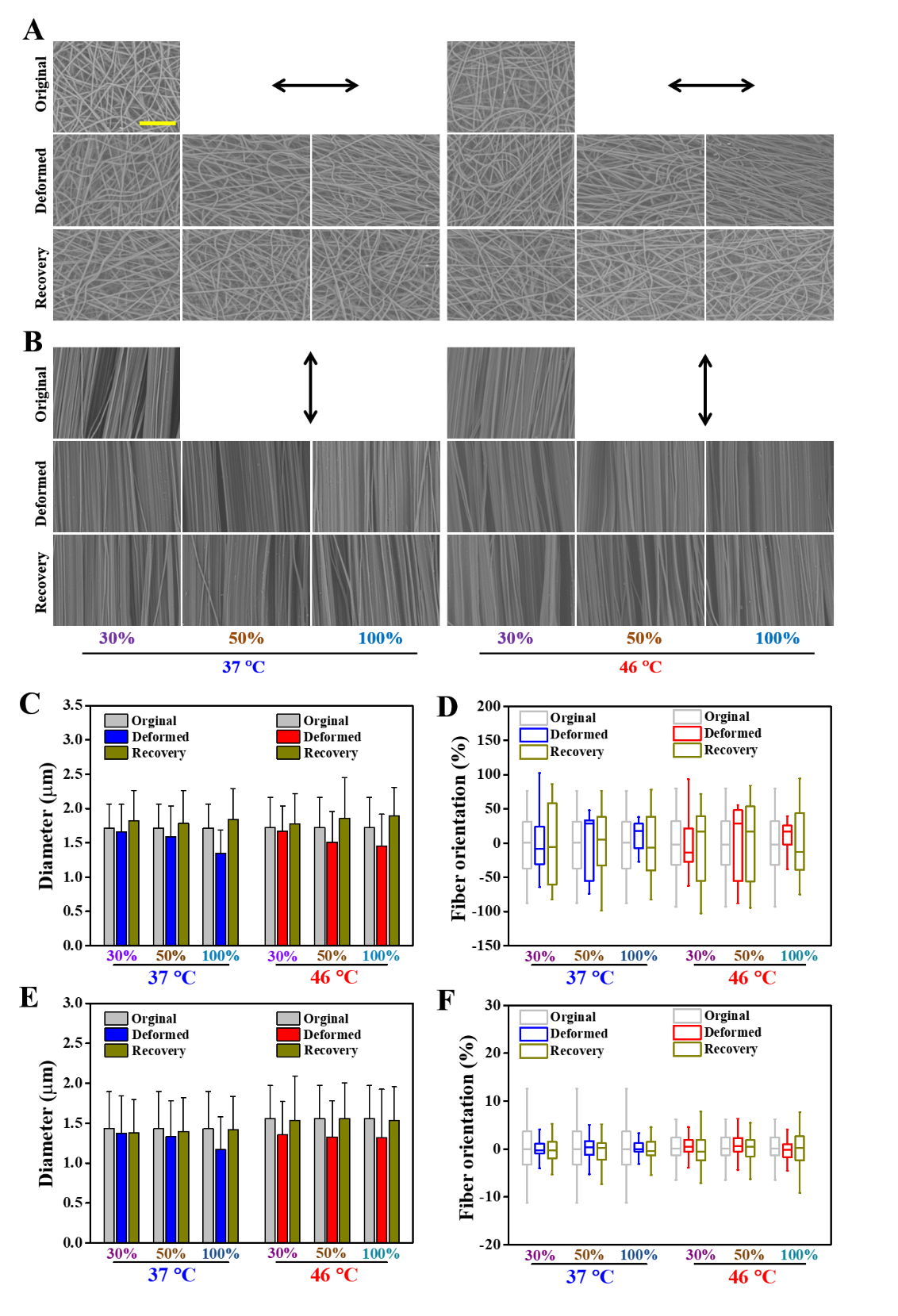


**Figure S****1.** Comparative study on morphology changes regulated by shape-programming parameters (i.e., *T_prog_* and *ε_deform_*) between random fibers and aligned fibers. (a) SEM images of Random, scale bare = 20 μm. (b) SEM images of Aligned. (c) Quantified diameter changes in the Random. (d) Quantified fiber orientation changes in the Random. (e) Quantified diameter changes in the Aligned. (f) Quantified fiber orientation changes in the Aligned.


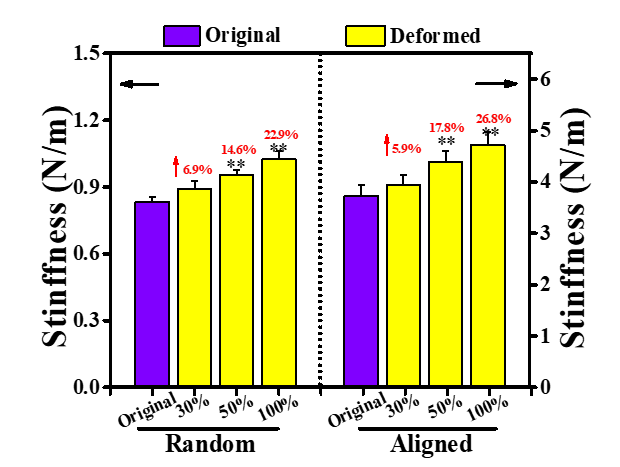


**Figure S2.** Stiffness of the fibrous samples derived from **Figure 3 (g, h)** by having the elastic modulus multiplied with cross-sectional area and divided with gauge length of the tested samples

**Table S1** The *D_r_* values determined from P-FTIR for different fibrous mats

| Wave number (cm^−1^) | Sample | A_//_ | A_⊥_ | *D_r_* = A_//_/A_⊥_ |
| --- | --- | --- | --- | --- |
| 1086 | Random | 0.30 | 0.31 | 0.97 |
|  | Aligned | 0.24 | 0.26 | 0.92 |
| 1179 | Random | 0.46 | 0.47 | 0.98 |
|  | Aligned | 0.45 | 0.59 | 0.76 |
| 1759 | Random | 0.27 | 0.27 | 1.00 |
|  | Aligned | 0.37 | 0.26 | 1.42 |
| 1722 | Random | 0.33 | 0.36 | 0.92 |
|  | Aligned | 0.21 | 0.25 | 0.84 |
